# Prevalence of Non-diabetic Hyperglycemia in Young Adults and Its Impact on Periodontal Health

**DOI:** 10.7759/cureus.53847

**Published:** 2024-02-08

**Authors:** Ali Mohammad Alhomaid, Muzammil Moin Ahmed

**Affiliations:** 1 Dental Hygiene, Qassim Regional Dental Centre, Buraidah, SAU; 2 Dental and Oral Health, College of Applied Health Sciences in Ar Rass, Qassim University, Ar Rass, SAU

**Keywords:** risk factor., prevalence, periodontal medicine, diabetes, periodontitis, prediabetes, non-diabetic hyperglycaemia

## Abstract

Background

Non-diabetic hyperglycemia is a transitional phase of hyperglycemia that poses a hidden risk for the development of diabetes mellitus and related complications, including periodontal destruction. The current study sought to determine the prevalence of non-diabetic hyperglycemia in young adults and any possible links to periodontal health.

Methods

A total of 400 participants in this cross-sectional study were evaluated for non-diabetic hyperglycemia between the ages of 18 and 35 years. Group I consisted of non-diabetic hyperglycemic participants. Group II comprised an equal number of matched, healthy subjects. The groups' hyperglycemic and clinical periodontal characteristics were contrasted. Using a one-sample t-test and logistic regression analysis, the acquired data were subjected to statistical analysis.

Results

The prevalence of non-diabetic hyperglycemia was 19%, with men (13%) having a higher prevalence than women (6%). The mean fasting plasma glucose and hemoglobin A1c (HbA1c) levels were 114.47 ± 6.40 mg/dL and 6.10 ± 0.21%, respectively, for group I, and 85.72 ± 7.24 mg/dL and 4.38 ± 0.70% for group II. When compared to healthy controls, all periodontal parameters, including plaque index, gingival index, bleeding on probing, probing depth, and clinical attachment loss, were significantly higher in group I non-diabetic hyperglycemic patients. The regression analysis revealed statistically significant links between hyperglycemic and periodontal parameters.

Conclusion

The prevalence of non-diabetic hyperglycemia among young adults is a serious concern similar to that of older adults with the risk for periodontal diseases. Non-diabetic hyperglycemic considerations in young adults should be emphasized in dental and medical clinics to reduce the risk of developing diabetes mellitus and to avoid irreversible periodontal tissue damage.

## Introduction

Non-diabetic hyperglycemia, also referred to as prediabetes, is a condition characterized by hyperglycemia, which is too rare to be considered diabetes mellitus. It was an intermittent metabolic state prior to the development of diabetes. The American Diabetes Association (ADA) has defined non-diabetic hyperglycemia as impaired fasting glucose of 100-125 mg/dL and impaired glucose tolerance of 140-199 mg/dL with hemoglobin A1c (HbA1c) of 5.7% to 6.4% [1]. According to the Centers for Disease Control and Prevention, 38% of United States adults aged over 18 years have non-diabetic hyperglycemia [2]. Non-diabetic hyperglycemia, according to the International Diabetes Federation, "presents a significant concern for future diabetes risk worldwide." [3]. Non-diabetic hyperglycemia is a risk factor for developing type 2 diabetes mellitus and associated microvascular and cardiometabolic complications [3-5]. It increases the pro-inflammatory biomarkers [6,7] and alters the microbiota [8], which may predispose to the development of microbial diseases.

Periodontitis, a plaque-induced multifactorial dental disease affecting the teeth’s supporting structures, is considered the sixth complication of diabetes mellitus, and both of these diseases share bilateral relationships that negatively affect their course and control [9,10]. A systematic review update published by the American Academy of Periodontology (AAP) and the European Federation of Periodontology (EFP) confirms that periodontitis increases the risk of poor glycemic control and related complications [11]. According to AAP and EFP, diabetes is a significant risk factor for periodontal attachment loss, with elevated HbA1c levels in the periodontitis grading system in the most recent classification of periodontal diseases and conditions [12], indicating poor glycemic control. Though plaque-generated host-inflammatory conflict is the primary etiological reason for the onset of periodontal disease, the periodontal tissue changes brought about by the hyperglycemic state exaggerate the severity of periodontal diseases. The constant hyperglycemic state results in the formation and accumulation of advanced glycation end products, leading to the stimulated production of proinflammatory cytokines and components that mediate periodontal destruction. Hyperglycemia and associated compromised host immune mechanisms result in microbial dysbiosis, favoring the shift toward anaerobic and tissue-invasive periodontal pathogens. Chronic hyperglycemia induces structural and functional alterations in periodontal tissues, including the epithelium, connective tissue, and collagen metabolism. All of these manifest with clinical signs of periodontal disease, including bleeding on probing (BOP), increased periodontal probing depths, clinical attachment loss (CAL), and subsequent bone and tooth loss.

Despite the availability of enormous literature about the association between diabetes mellitus and periodontal diseases, data regarding the association between non-diabetic hyperglycemia and periodontitis are limited, with a lack of consensus among the researchers. The periodontal parameters are increased in non-diabetic hyperglycemic patients, as reported in the studies by Abduljabbar et al. and Javed et al. [13,14]. Another study by Kowall et al. has shown no association between clinical periodontal parameters and non-diabetic hyperglycemia [15]. Also, the data on the prevalence of non-diabetic hyperglycemia is insufficient.

Considering the cruciality of the non-diabetic hyperglycemic problem, the scarcity of scientific literature about its prevalence in young adults, and the lack of consensus about the role of non-diabetic hyperglycemia in periodontal disease severity, the present study was planned to assess the prevalence of non-diabetic hyperglycemia in young adults and its potential association with periodontal health.

## Materials and methods

The subjects in this cross-sectional descriptive study ranged in age from 18 to 35 years. The study cohorts were randomly chosen from screenings conducted at primary medical health centers, shopping centers, and gyms. The study protocol is as per the updated Declaration of Helsinki with the approval of the ethical clearance committee. Every participant was informed of the study's goal and their part in it, and their informed consent was obtained.

The 400 subjects meeting the inclusion and exclusion criteria were screened for non-diabetic hyperglycemia as per the diagnostic guidelines of the ADA. Of the 400 subjects screened, 76 non-diabetic hyperglycemic-diagnosed subjects were included in group I (the non-diabetic hyperglycemic group), and an equal number of 76 non-diabetic hyperglycemic subjects were randomly allotted to group II (the healthy group) based on the inclusion criteria. Computer-generated simple randomization was used for group II.

The inclusion criteria of the study consisted of the following: 1) for group I, patients with a fasting plasma glucose (FPG) concentration of ≥100 mg/dL but ≤125 mg/dL and HbA1c values of ≥5.7% to ≤6.4%, and for group II, patients with an FPG concentration of <100 mg/dL and HbA1c <5.7%; 2) patients not on antimicrobial therapy within the previous three months; and 3) patients with normal weight, i.e., BMI between 18.5 and 24.9 kg/m^2^. The exclusion criteria consisted of the following: 1) subjects below the age of 18 and above 35 years; 2) individuals with a history of any diseases, conditions, and/or medications that influence the periodontium; 3) patients who have undergone periodontal therapy in the last six months; 4) current and former tobacco and alcohol users; and 5) pregnant and other medically compromised individuals.

A detailed history was recorded for each patient, including demographic information, a family history of diabetes, and a history of deleterious tobacco habits. Then the thorough periodontal examination was carried out together by trained periodontists and dental hygienists, which included the recording of the sickness and Loe plaque index (PI), the Loe-Silness gingival index (GI), BOP, probing depth (PD), and CAL. Six sites per tooth were recorded for BOP, PD, and CAL using the University of North Carolina graduated probe (UNC-15). The highest PD and CAL values per tooth were considered for calculations.

For evaluating non-diabetic hyperglycemic status, all participants were asked to fast for 10 hours before their next appointment. To avoid limitations from the FPG assessment and to have a more confirmatory test, all the subjects underwent both FPG and HbA1c analysis in the laboratories of Ar Rass primary health centers. Patients with FPG concentrations of ≥100 mg/dL but ≤125 mg/dL and HbA1c values between 5.7% and 6.4% were considered non-diabetic hyperglycemic (group I).

Statistical analysis

The data thus collected were tabulated and subjected to further statistical analysis using IBM SPSS Version 18.0 software (IBM Corp., Armonk, NY). The two groups were compared using the mean, median, and standard deviation for continuous variables and the frequency and percentage for categorical variables. A one-sample t-test and regression analysis were performed to compare quantitative variables between the two groups. The qualitative variables were compared using the chi-squared test. A p-value of <0.05 is identified as statistically significant.

## Results

Among the 400 subjects examined, 48.25% (n = 193) were women, and 51.75% (n = 207) were men. The mean age for subjects was 27.50 ± 6.23 years. The prevalence of non-diabetic hyperglycemia among young adults was 19% (n = 76), with a higher prevalence in men (13%, n = 52) than in women (6%, n = 24). The prevalence in subjects aged <30 years and ≥30 years was 10% (n = 40) and 9% (n = 36), respectively. The comparison of various periodontal clinical parameters, such as FPG and HbA1c, among the two study groups is shown in Table 1.

**Table 1:** Comparison of various parameters among two study groups using a one-sample t-test BOP, bleeding on probing; CAL, clinical attachment loss; FPG, fasting plasma glucose; GI, gingival index; HbA1c, hemoglobin A1C (glycated hemoglobin); M, men; PD, probing depth; PI, plaque index; W, women

Parameters	Group I		Group II		p-Value
	Mean	SD	Mean	SD	
Mean age	28.0395	6.15671	27.9868	6.31927	0.165
Gender	52/M	24/W	42/M	34/W	0.05
FPG	114.4737	6.40723	85.7237	7.24541	0.028
HbA1c	6.1079	0.21277	4.3868	0.70962	0.001
PI	2.1171	0.66320	1.2368	0.70462	0.003
GI	1.6750	0.80384	0.9342	0.69887	0.045
BOP	83.5395	16.93630	51.2368	24.52094	0.008
PD	5.3566	1.89035	2.6158	1.27976	0.006
CAL	5.2983	2.40639	1.5007	2.26098	0.004

The values of all the periodontal parameters, including PI, GI, BOP, PD, and CAL, were significantly higher (p < 0.05) in group I patients compared to group II controls. The logistic regression analysis showing the correlation of FPG and HbA1c titers with various periodontal parameters in two study groups is depicted in Table 2.

**Table 2:** Regression analysis showing the relationship of FPG and HbA1c with various parameters in two study groups BOP, bleeding on probing; CAL, clinical attachment loss; FPG, fasting plasma glucose; GI, gingival index; HbA1c, hemoglobin A1C (glycated hemoglobin); PD, probing depth; PI, plaque index

Model	Group I					Group II					All study subjects (both groups)					
	Unstandardized coefficients		Standard coefficient	t	p-Value	Unstandardized coefficients		Standard coefficient	t	p-Value	Unstandardized coefficients		Standard coefficient	t	p-Value	
	β	Standard error	β			β	Standard error	β			β	Standard error	β			
Relationship of FPG with various parameters															
Constant	98.09	0.808	-	121.362	0.001	76.829	2.650	-	28.996	0.000	66.872	2.337	-	28.611	0000	
PI	2.847	0.528	0.295	5.389	0.001	2.895	1.483	0.282	1.952	0.055	5.898	1.748	0.300	3.374	0.001	
GI	4.529	0.530	0.568	8.553	0.001	1.168	1.372	0.113	1.851	0.037	-4.621	1.565	-0.243	-2.953	0.004	
BOP	1.004	0.512	0.410	2.021	0.049	3.113	1.042	0.383	2.710	0.008	0.230	0.046	0.383	5.056	0.001	
PD	3.331	0.236	0.908	1.402	0.015	7.118	1.990	0.021	1.120	0.005	2.948	0.922	0.391	3.198	0.002	
CAL	7.127	0.146	0.408	0868	0.008	2.606	1.581	0.189	1.044	0.010	2.616	1.124	0.412	1.672	0.003	
Relationship of HbA1c with various parameters															
Constant	5.533	0.035	-	159.521	0.001	3.505	0.258	-	13.589	0.000	3.274	0.168	-	19.459	0.001	
PI	0.088	0.023	0.275	3.888	0.001	0.299	0.144	0.297	2.071	0.042	0.401	0.126	0.323	3.187	0.002	
GI	0.128	0.023	0.485	5.650	0.001	-0.118	0.134	-0.116	2.484	0.030	-0.417	0.113	-0.346	-3.701	0.001	
BOP	0.001	0.001	0.049	1.209	0.031	0.011	0.004	0.381	2.708	0.008	0.019	0.003	0.492	5.712	0.001	
PD	0.019	0.010	0.165	1.829	0.021	-0.011	0.096	-0.020	1.518	0.007	3.2881	0.066	0.153	1.101	0.023	
CAL	0.004	0.006	0.048	0.682	0.049	0.058	0.057	6.104	0.023	0.010	9.126	0.046	0.255	1.876	0.036	

It was observed that in both the study groups and all study subjects, statistically significant (p < 0.05) relationships exist between hyperglycemic parameters and periodontal parameters. The relationship of FPG and HbA1c with various periodontal parameters in both genders and in patients with <30 years and ≥30 years of age is shown in Table 3.

**Table 3:** Regression analysis showing the relationship of FPG and HbA1c with various parameters in females and males aged <30 and ≥30 years BOP, bleeding on probing; CAL, clinical attachment loss; FPG, fasting plasma glucose; GI, gingival index; HbA1c, hemoglobin A1C (glycated hemoglobin); PD, probing depth; PI, plaque index

Model	Women					Men					<30 years					≥30 years				
	Unstandardized coefficients		Standard coefficient	t	p-Value	Unstandardized coefficients		Standard coefficient	t	p-Value	Unstandardized coefficients		Standard coefficient	t	p-Value	Unstandardized coefficients		Standard coefficient	t	p-value
	β	Standard error	β			β	Standard error	β			β	Standard error	β			β	Standard error	β		
Relationship of FPG with various parameter																				
Constant	70.353	4.348	-	16.180	0.001	65.241	2.853	-	22.869	0.001	69.917	3.162	-	22.113	0.001	63.895	3.436	-	18.598	0.001
PI	6.146	3.107	0.333	1.978	0.053	6.256	2.174	0.306	2.878	0.005	1.635	2.148	0.079	0.761	0.049	11.304	2.727	0.584	4.146	0.001
GI	-3.411	3.127	-0.173	-1.091	0.020	-5.741	1.802	-0.307	-3.186	0.002	-3.320	1.886	-0.153	-1.761	0.002	-5.850	2.564	-0.322	-2.281	0.026
BOP	0.213	0.080	0.366	2.669	0.010	0.221	0.057	0.359	3.898	0.001	0.256	0.058	0.448	4.415	0.001	0.165	0.071	0.255	2.317	0.024
PD	0.862	1.693	0.105	0.509	0.013	4.342	1.126	0.601	3.857	0.001	2.781	1.202	0.314	2.313	0.023	2.758	1.468	0.375	1.878	0.045
CAL	1.650	1.107	0.303	1.490	0.042	-0.069	0.792	-0.013	-.088	0.030	1.575	0.806	0.277	1.954	0.054	0.317	1.081	0.054	0.293	0.050
Relationship of HbA1c with various parameter																				
Constant	3.471	0.299	-	11.606	0.001	3.161	0.214	-	14.750	0.001	3.335	0.258	-	12.933	0.001	03.225	0.230	-	14.021	0.001
PI	0.417	0.214	0.344	1.952	0.056	0.412	0.163	0.324	2.524	0.013	0.206	0.175	0.145	5.179	0.042	0.642	0.183	0.579	3.518	0.001
GI	-0.304	0.215	-0.235	2.415	0.013	-0.499	0.135	-0.428	-3.688	0.001	-0.338	0.154	-0.228	-2.200	0.031	-0.457	0.172	-0.438	-2.659	0.010
BOP	0.017	0.006	0.456	3.166	0.003	0.018	0.004	0.482	4.340	0.001	0.020	0.005	0.520	4.298	0.001	0.014	0.005	0.379	2.947	0.005
PD	-0.050	0.116	-0.092	1.905	0.016	0.165	0.085	0.367	1.951	0.054	0.081	0.098	0.134	1.826	0.011	0.081	0.098	0.191	2.820	0.015
CAL	0.147	0.076	0.412	1.931	0.059	0.040	0.059	0.124	2.172	0.003	0.124	0.066	0.320	3.991	0.024	0.058	0.072	0.173	9.002	0.026

Regarding FPG, the PI, GI, BOP, and PD values were significantly (p < 0.05) higher in men, while the CAL value was higher among women. In contrast, the BOP, PD, and CAL values were significantly higher in patients <30 years of age, while the PI and GI scores were higher in patients aged ≥30 years. Regarding HbA1c, the GI, BOP, and PD values were higher in men, while PI and CAL values were higher in women. On the contrary, BOP and CAL values were noted more in patients aged <30 years, while PI and GI scores were higher in patients aged ≥30 years, and no difference was observed in PD.

## Discussion

The ADA and International Diabetes Foundation express concern about non-diabetic hyperglycemia and its prevalence and complications in adults. However, non-diabetic hyperglycemic prevalence among young adults remains scantily addressed. The present study attempted to explore the prevalence of non-diabetic hyperglycemia in young adults and assess its influence on periodontal status, which was also in its initial phase of establishment. Our study revealed a 19% non-diabetic hyperglycemic prevalence rate among young adults. This prevalence among young adults is not very different from that of older adults, which has been reported at 26% by Shang et al. [16]. Presently, only two studies, one by Andes et al. and one by Ben Haider and Ziyab, are available for comparison of our prevalence data for young adults. Andes et al. reported a 24% prevalence among young adults in the US, which is higher than our study results, and Ben Haider and Ziyab reported a lower prevalence of 6.3% in the Kuwaiti population [17,18]. The influence of race or ethnicity cannot be disregarded in the comparison of these prevalence rates. Although a Saudi Arabian study by Aldossari et al. has reported a higher prevalence of 27% than our study, it did not consider the female population in its study without emphasis on young adults [19]. Our study results also showed comparatively higher non-diabetic hyperglycemia in men than women, which is in equivalency with the finding reported by Andes et al. [17]. The higher predilection in men could be because risk factors for progression toward prediabetes, including deleterious habits, a strenuous daily lifestyle, etc., were mainly seen in males, or it might have been male predilection, which needs further longitudinal investigation. The mean age of non-diabetic hyperglycemic patients was 28.03 ± 6.15 years in our study, with a similar trend observed in studies reported in the US [20] and China [21]. The prevalence of non-diabetic hyperglycemia among young adults in our study is contrary to the consideration of age 45 years or older as a risk by the ADA, which warrants further research in this aspect, and our study may form foundational evidence for this. The findings of our study are also in contradiction with the studies by Salmeron et al. and Hashemi et al., which suggest a risk of non-diabetic hyperglycemia in the older age group [22,23]. Moreover, this remarkable finding of our study gives us a message to initiate preventive and therapeutic measures and health educational programs on non-diabetic hyperglycemia at an early age to prevent our young population from the vast health effects and later complications of this disease. If non-diabetic hyperglycemia is left untreated, 15% to 30% of people with it progress to type 2 diabetes mellitus within five years [24]. FPG and HbA1c analyses were utilized to assess the non-diabetic hyperglycemic status of study subjects. A study by Ghazanfari et al. demonstrated FPG as a more reliable test to assess hyperglycemia [25]. According to an Irish study by Sinnott et al., using FPG as initial screening may underestimate the prevalence of hyperglycemia [26]. Hence, a combination of FPG and HbA1c was used to enhance the diagnostic accuracy in our study, as indicated by Sherwani et al. [27].

Many studies postulate different mechanistic pathways to explain possible links between established diabetes mellitus and periodontal disease [28-30]. Diabetic patients have a higher risk for periodontitis development, probably due to vascular changes, neutrophil dysfunction, altered systemic inflammatory responses, altered collagen synthesis, microbiotic factors, or genetic predisposition. In contrast, the potential biological mechanisms explaining the association between non-diabetic hyperglycemia (prediabetes) and periodontal status have been scarcely studied. It is important to assess the early stages to better understand the natural history and interrelationships between the two diseases. Previous studies have assessed the potential role of reactive oxygen species in the association [31,32]. Impaired glycemic status is associated with an increased production and accumulation of reactive oxygen species in the body tissues, including the periodontium.

The present study reported significant correlations between various clinical parameters of periodontitis (PI, GI, BOP, PD, and CAL) and FPG and HbA1c levels in non-diabetic hyperglycemic patients. Similar results were noted by Abduljabbar et al. [13] and Javed et al. [13], whereas the study of Kowall et al. [15] contradicts this finding. Elevated blood sugar promotes the growth of aggressive periodontal bacteria such as *Porphyromonas gingivalis*, *Prevotella intermedia*, *Tannerella forsythus*, and *Aggregatibacter (Actinobacillus)* and induces structural and immunological changes in the periodontal tissue that cause destruction [33,34], which, in turn, clinically manifests as increased periodontal parameter values. The occurrence and burden of periodontitis among the elderly stay high [35]. A study by Eke et al. indicates a higher prevalence of periodontal disease in people over the age of 30 years [36]. In our study, the observation of increased periodontal destruction indicated by higher values of periodontal parameters in young adults strongly suggests an influence of non-diabetic hyperglycemia. In addition, shared risk factors such as tobacco use, obesity, systemic disease, periodontal medications, and diabetes were excluded in this study. This was not seen in other correlative studies, indicating an independent role of non-diabetic hyperglycemia in the etiology of periodontitis.

However, this study has certain limitations such as the small sample size and the lack of use of radiographs for the diagnosis of periodontal disease. Therefore, long-term studies with large sample sizes are needed to support our findings. More research is needed on the prevalence of non-diabetic hyperglycemia, with a focus on young adults.

## Conclusions

In the current study, the prevalence of non-diabetic hyperglycemia in young adults is 19%, with a higher prevalence in males. The results of this study confirm that the non-diabetic hyperglycemic condition is associated with periodontal inflammation. Hyperglycemia in non-diabetic individuals can increase the severity of periodontal parameters. Early diagnosis of non-diabetic hyperglycemia and appropriate measures to prevent it are of fundamental importance to reduce the risk of developing diabetes mellitus and avoid largely irreversible periodontal tissue damage. The authors, therefore, recommend that clinicians and public health policymakers give immediate attention to non-diabetic hyperglycemia, especially in young adults. Non-diabetic hyperglycemia screening should also be considered as a protocol prior to initiating periodontal treatment similar to diabetes.

## References

[1] American Diabetes Association (2014). diagnosis and classification of diabetes mellitus. Diabetes Care.

[2] (2023). Centers for Disease Control and Prevention. National Diabetes Statistics Report. https://www.cdc.gov/diabetes/data/statistics-report/index.html.

[3] (2023). International Diabetes Federation IDF Diabetes Atlas. https://diabetesatlas.org/atlas/tenth-edition/.

[4] Pour OR, Dagogo-Jack S (2011). Prediabetes as a therapeutic target. Clin Chem.

[5] Grundy SM (2012). Pre-diabetes, metabolic syndrome, and cardiovascular risk. J Am Coll Cardiol.

[6] Zeng J, Xu Y, Shi Y, Jiang C (2018). Inflammation role in sensory neuropathy in Chinese patients with diabetes/prediabetes. Clin Neurol Neurosurg.

[7] Yang SJ, Hwang SY, Choi HY (2011). Serum selenoprotein P levels in patients with type 2 diabetes and prediabetes: implications for insulin resistance, inflammation, and atherosclerosis. J Clin Endocrinol Metab.

[8] Allin KH, Tremaroli V, Caesar R (2018). Aberrant intestinal microbiota in individuals with prediabetes. Diabetologia.

[9] Muzammil Muzammil, Jayanthi D, Faizuddin M, Noor Ahamadi HM (2017). Association of interferon lambda-1 with herpes simplex viruses-1 and -2, Epstein-Barr virus, and human cytomegalovirus in chronic periodontitis. J Investig Clin Dent.

[10] Löe H (1993). Periodontal disease: the sixth complication of diabetes mellitus. Diabetes Care.

[11] Graziani F, Gennai S, Solini A, Petrini M (2018). A systematic review and meta-analysis of epidemiologic observational evidence on the effect of periodontitis on diabetes. An update of the EFP-AAP review. J Clin Periodontol.

[12] Tonetti MS, Greenwell H, Kornman KS (2018). Staging and grading of periodontitis: framework and proposal of a new classification and case definition. J Periodontol.

[13] Abduljabbar T, Al-Sahaly F, Al-Kathami M, Afzal S, Vohra F (2017). Comparison of periodontal and peri-implant inflammatory parameters among patients with prediabetes, type 2 diabetes mellitus and non-diabetic controls. Acta Odontol Scand.

[14] Javed F, Tenenbaum HC, Nogueira-Filho G (2013). Periodontal inflammatory conditions among gutka chewers and non-chewers with and without prediabetes. J Periodontol.

[15] Kowall B, Holtfreter B, Völzke H, Schipf S, Mundt T, Rathmann W, Kocher T (2015). Pre-diabetes and well-controlled diabetes are not associated with periodontal disease: the SHIP Trend Study. J Clin Periodontol.

[16] Shang Y, Fratiglioni L, Marsegilia A (2018). Incidence and evolution of prediabetes among older adults—a population-based cohort study. Diabetes.

[17] Andes LJ, Cheng YJ, Rolka DB, Gregg EW, Imperatore G (2020). Prevalence of prediabetes among adolescents and young adults in the United States, 2005-2016. JAMA Pediatr.

[18] Ben Haider NY, Ziyab AH (2016). Prevalence of prediabetes and its association with obesity among college students in Kuwait: a cross-sectional study. Diabetes Res Clin Pract.

[19] Aldossari KK, Aldiab A, Al-Zahrani JM (2018). Prevalence of prediabetes, diabetes and its associated risk factors among males in Saudi Arabia: a population-based survey. J Diabetes Res.

[20] (2023). Centers for Disease Control and Prevention. National diabetes fact sheet: general information and national estimates on diabetes in the United States. https://stacks.cdc.gov/view/cdc/13329.

[21] Tian H, Song G, Xie H, Zhang H, Tuomilehto J, Hu G (2009). Prevalence of diabetes and impaired fasting glucose among 769,792 rural Chinese adults. Diabetes Res Clin Pract.

[22] Salmerón D, Gómez García F, Pons-Fuster E, Pérez-Sayáns M, Lorenzo-Pouso AI, López-Jornet P (2019). Screening for prediabetes and risk of periodontal disease. Diabetes Metab Syndr.

[23] Hashemi SJ, Karandish M, Cheraghian B, Azhdari M (2022). Prevalence of prediabetes and associated factors in southwest Iran: results from Hoveyzeh cohort study. BMC Endocr Disord.

[24] (2023). American Medical Association, Centers for Disease Control and Prevention. Preventing Type 2 Diabetes. Atlanta: Centers for Disease Control and Prevention. https://www.cdc.gov/diabetes/prevention/pdf/stat_toolkit.pdf..

[25] Ghazanfari Z, Haghdoost AA, Alizadeh SM, Atapour J, Zolala F (2010). A comparison of HbA1c and fasting blood sugar tests in general population. Int J Prev Med.

[26] Sinnott M, Kinsley BT, Jackson AD (2015). Fasting plasma glucose as initial screening for diabetes and prediabetes in Irish adults: the Diabetes Mellitus and Vascular health initiative (DMVhi). PLoS One.

[27] Sherwani SI, Khan HA, Ekhzaimy A, Masood A, Sakharkar MK (2016). Significance of HbA1c test in diagnosis and prognosis of diabetic patients. Biomark Insights.

[28] Manouchehr-Pour M, Spagnuolo PJ, Rodman HM, Bissada NF (1981). Impaired neutrophil chemotaxis in diabetic patients with severe periodontitis. J Dent Res.

[29] McMullen JA, Van Dyke TE, Horoszewicz HU, Genco RJ (1981). Neutrophil chemotaxis in individuals with advanced periodontal disease and a genetic predisposition to diabetes mellitus. J Periodontol.

[30] Seppälä B, Sorsa T, Ainamo J (1997). Morphometric analysis of cellular and vascular changes in gingival connective tissue in long-term insulin-dependent diabetes. J Periodontol.

[31] King GL, Loeken MR (2004). Hyperglycemia-induced oxidative stress in diabetic complications. Histochem Cell Biol.

[32] Ohnishi T, Bandow K, Kakimoto K, Machigashira M, Matsuyama T, Matsuguchi T (2009). Oxidative stress causes alveolar bone loss in metabolic syndrome model mice with type 2 diabetes. J Periodontal Res.

[33] Newman MG, Takei HH, Carranza FA (2019). Clinical Periodontology.

[34] Chang PC, Lim LP (2012). Interrelationships of periodontitis and diabetes: a review of the current literature. J Dent Sci.

[35] López R, Smith PC, Göstemeyer G, Schwendicke F (2017). Ageing, dental caries and periodontal diseases. J Clin Periodontol.

[36] Eke PI, Dye BA, Wei L, Thornton-Evans GO, Genco RJ (2012). Prevalence of periodontitis in adults in the United States: 2009 and 2010. J Dent Res.

